# R-Ras1 and R-Ras2 Expression in Anatomical Regions and Cell Types of the Central Nervous System

**DOI:** 10.3390/ijms23020978

**Published:** 2022-01-17

**Authors:** Gonzalo Garcia-Martin, Miriam Sanz-Rodriguez, Berta Alcover-Sanchez, Marta P. Pereira, Francisco Wandosell, Beatriz Cubelos

**Affiliations:** 1 Departamento de Biología Molecular, Universidad Autónoma de Madrid, 28049 Madrid, Spain; gonzalo.garciamartin@estudiante.uam.es (G.G.-M.); mithrelas88@googlemail.com (M.S.-R.); berta.alcover@cbm.csic.es (B.A.-S.); pereiram@cbm.csic.es (M.P.P.); 2 Centro de Biología Molecular Severo Ochoa, Universidad Autónoma de Madrid, 28049 Madrid, Spain; fwandosell@cbm.csic.es; 3 Alzheimer’s Disease and Other Degenerative Dementias, Centro de Investigación Biomédica en Red de Enfermedades Neurodegenerativas (CIBERNED), 28031 Madrid, Spain

**Keywords:** myelin, R-Ras, optic nerve, neurodegenerative diseases, neuron

## Abstract

Since the optic nerve is one of the most myelinated tracts in the central nervous system (CNS), many myelin diseases affect the visual system. In this sense, our laboratory has recently reported that the GTPases R-Ras1 and R-Ras2 are essential for oligodendrocyte survival and maturation. Hypomyelination produced by the absence of one or both proteins triggers axonal degeneration and loss of visual and motor function. However, little is known about R-Ras specificity and other possible roles that they could play in the CNS. In this work, we describe how a lack of R-Ras1 and/or R-Ras2 could not be compensated by increased expression of the closely related R-Ras3 or classical Ras. We further studied R-Ras1 and R-Ras2 expression within different CNS anatomical regions, finding that both were more abundant in less-myelinated regions, suggesting their expression in non-oligodendroglial cells. Finally, using confocal immunostaining colocalization, we report for the first time that R-Ras2 is specifically expressed in neurons. Neither microglia nor astrocytes expressed R-Ras1 or R-Ras2. These results open a new avenue for the study of neuronal R-Ras2’s contribution to the process of myelination.

## 1. Introduction

The neural structures and circuits that receive, transform, ship, and integrate the light signals that allow human beings to see are deeply complex; information must be received by the retinal ganglion cells (RGCs), which then project their axons through the optic nerve (ON), decide whether to cross the optic chiasm, and arrive at the lateral geniculate nucleus. From there, new fibers emerge to connect with the primary visual cortex, where information is correctly integrated to form images. In some diseases—such as Leber’s hereditary optic neuropathy, or some subtypes of Charcot–Marie–Tooth disease—neurons are predominantly affected [1,2]. However, neurons are not the only cell type involved in the delivery of visual information.

Myelin, produced in the central nervous system (CNS) by oligodendrocytes (OLs), is critically required in the vertebrate nervous system in order to enable fast and efficient synaptic transmission [3]. In this sense, many demyelinating diseases of the CNS disturb the visual system. Neuromyelitis optica (Devic’s disease) is an inflammatory demyelinating disease that preferentially affects the ON and spinal cord (SC) [4]. Initially described as a variation of multiple sclerosis (MS), it is now classified as a distinct disease due to the presence of autoantibodies against aquaporin-4 water channels or myelin oligodendrocyte glycoprotein [5]. For instance, one of the clinical manifestations of MS is optic neuritis, which is an inflammatory optic neuropathy that can affect one or both ONs [6]. While some of these diseases are spontaneous and appear in later stages of life, others—such as hypomyelinating leukodystrophies—appear during infancy or early childhood, as they are associated with neurodevelopmental defects [7,8]. Because of their high complexity and low prevalence, there is a lack of research and effective treatments for most demyelinating diseases. Therefore, it is essential to deepen our knowledge of the molecular mechanisms involved in the myelination process.

Myelination is a multistep process that requires the interaction between neurons and OLs [9,10,11,12]. To achieve successful myelination, OLs need both intracellular and extracellular signals to contact the axons and generate the myelin sheaths. After differentiating from OL progenitor cells (OPCs), myelinating OLs extend their processes to wrap around axons and form myelin sheaths [13,14,15]. In this way, neuronal axons are metabolically supported and protected from the extracellular space [16,17,18]. Two intracellular signaling pathways are involved in the different steps of the myelination process: PI3K/Akt/mTOR, and Erk-MAPK [19,20]. Even though each pathway has different roles, they both act in concert to ensure effective myelination. One interesting upstream target of these pathways is the Ras superfamily of proteins [21], which act as molecular switches that can transduce extracellular signals to intracellular signaling, thereby regulating proliferation, differentiation and cell survival [22,23]. The Ras superfamily of proteins comprises more than 150 small GTPases subdivided into 5 major families according to their homology [24]; of these, the Ras-related (R-Ras) subfamily shares the highest sequence identity (52–55%) with the classical H-Ras, K-Ras, and N-Ras. The R-Ras subfamily is composed of R-Ras1 (R-Ras), R-Ras2 (TC21), and R-Ras3 (M-Ras), but unlike classical Ras, their functional implications have been less studied [25]. R-Ras1 encodes a 218-amino-acid protein (23.5 kDa) that shares 55% sequence identity with H-Ras, 65% with R-Ras2, and 46% with R-Ras3, while R-Ras2 encodes a 204-amino-acid protein (23.4 kDa) and R-Ras3 encodes a 208-amino-acid protein (23 kDa). It is known that expression of both R-Ras1 and R-Ras2 is ubiquitous, whereas R-Ras3 expression is more restricted to the CNS [21,26,27].

Although R-Ras3 has been considered the most important R-Ras of the brain [27,28,29], recent work in our laboratory has shown that R-Ras1 and R-Ras2 are essential for myelination of the principal myelinated tracts in the CNS—especially the ON [30]. The absence of R-Ras proteins in OLs from *R-Ras1^−/−^* and/or *R-Ras2^−/−^* mice resulted in different degrees of hypomyelination in the CNS, due to deficits in the OL maturation process. Specifically, *R-Ras1^−/−^* mice showed thinner myelin sheaths, while in the *R-Ras2^−/−^* mice there was a lower number of total myelinated axons. The hypomyelinating phenotype was worsened in the *R-Ras1^−/−^*;*R-Ras2^−/−^* mice, with up to 80% unmyelinated axons in the ON [30]. Subsequent work revealed that the lack of R-Ras proteins provoked alterations in the interactions between OLs and neurons. Aberrant myelination due to OL immaturity produced compensatory changes in axonal physiology, but these compensatory mechanisms were not enough to sustain axonal function over the long term, resulting in severe axon injury, degeneration, and loss of visual function [31]. These studies highlight the key role of R-Ras1 and -2 in OL maturation and the correct transmission and integration of visual function through their interaction with axons. For this reason, R-Ras mice were proposed as neurological models for the study of neurodegenerative diseases.

Little is known about the pattern of R-Ras1 and R-Ras2 expression in other CNS anatomical regions, or how their expression in other cell types may affect visual function. Here, we analyzed the pattern of expression of R-Ras1 and R-Ras2 in a variety of CNS anatomical regions, and found that those tissues with the highest expression of R-Ras1 and R-Ras2 were not the most highly myelinated ones. Furthermore, we found that an absence of R-Ras1 and R-Ras2 cannot be compensated by either R-Ras3 or the classical Ras members. Finally, we describe for the first time that, much like R-Ras1, R-Ras2 is specifically expressed in neurons, but not in astrocytes or microglia. This study opens a new line of research on the role of neuronal R-Ras2 function in the visual system.

## 2. Results

### 2.1. Loss of R-Ras1 and R-Ras2 Cannot Be Compensated by Either R-Ras3 or Classical Ras

**Figure 1:**
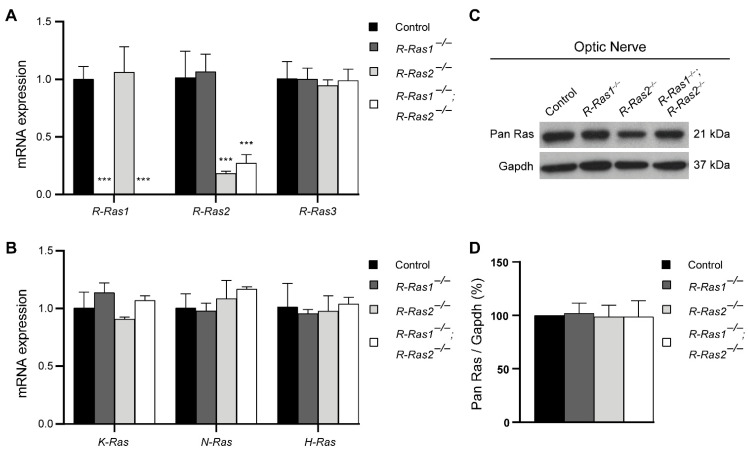
Absence of R-Ras1 and/or R-Ras2 does not modify R-Ras3 or classical Ras expression. (**A**) RT-qPCR experiments display relative expression of *R-Ras1*, *R-Ras2*, and *R-Ras3* in optic nerves from control, *R-Ras1^−/−^*, *R-Ras2^−/−^*, and *R-Ras1^−/−^*;*R-Ras2^−/−^* adult mice (P90). R-Ras1 mRNA expression showed significant differences in the *R-Ras1^−/−^* (*** *p* < 0.001) and *R-Ras1^−/−^;R-Ras2^−/−^* mice (*** *p* < 0.001). R-Ras2 mRNA expression showed significant differences in the *R-Ras2^−/−^* (*** *p* < 0.001) and *R-Ras1^−/−^*;*R-Ras2^−/−^* mice (*** *p* < 0.001). No significant differences were observed in the expression of R-Ras3 in the mutants relative to controls. (**B**) RT-qPCR experiments display relative expression of *K-Ras*, *N-Ras*, and *H-Ras* in ONs from control, *R-Ras1^−/−^*, *R-Ras2^−/−^*, and *R-Ras1^−/−^*;*R-Ras2^−/−^* adult mice (P90). No significant differences were observed in the expression of any classical Ras in the mutants relative to controls. (**C**) Western blot of classical Ras in ON lysates from adult (P90) mutant and control mice showed no significant differences. (**D**) Quantification of classical Ras levels normalized to GAPDH demonstrated no significant differences between the mutant and control mice. Bar graph represents the mean ± SD of the change as a percentage, relative to the control measurements. A two-tailed Student’s *t*-test was used for statistical analysis. GAPDH: glyceraldehyde 3-phosphate dehydrogenase; ON: optic nerve; SD: standard deviation. *n* = 3.

Traditionally, R-Ras3 has been considered to be the most important R-Ras of the CNS [27,28,29]. However, recent work from our laboratory has demonstrated that the lack of R-Ras1 and R-Ras2 provokes a severe myelin loss that compromises visual and motor functions [30,31]. In order to determine whether R-Ras1 and/or R-Ras2 function are specific or, rather, could be compensated by R-Ras3, we performed RT-qPCR experiments on ONs from *R-Ras1^−/−^*, *R-Ras2^−/−^*, and *R-Ras1^−/−^*;*R-Ras2^−/−^* adult mice relative to controls. R-Ras1 mRNA expression was undetectable in the *R-Ras1^−/−^* (6.39 × 10^−5^ ± 1.40 × 10^−5^, *p* < 0.001) and the *R-Ras1^−/−^*;*R-Ras2^−/−^* (5.22 × 10^−5^ ± 4.33 × 10^−6^, *p* < 0.001) mice relative to controls (1.00 ± 0.11), although no significant differences were observed in the *R-Ras2^−/−^* mice (1.06 ± 0.22). On the other hand, R-Ras2 mRNA expression was significantly reduced in the *R-Ras2^−/−^* (0.18 ± 0.02, *p* < 0.001) and *R-Ras1^−/−^*;*R-Ras2^−/−^* (0.27 ± 0.07, *p* < 0.001) mice relative to controls (1.02 ± 0.22). No significant differences were observed between the *R-Ras1^−/−^* (1.07 ± 0.15) and control mice. Lastly, for R-Ras3 we observed no significant changes in mRNA expression levels in *R-Ras1^−/−^* (1.00 ± 0.09), *R-Ras2^−/−^* (0.94 ± 0.05), or *R-Ras1^−/−^*;*R-Ras2^−/−^* mice (0.99 ± 0.10) relative to controls (1.01 ± 0.15) (Figure 1A). These results show that, despite the high degree of homology shared among R-Ras family members, loss of R-Ras1 and/or R-Ras2 cannot be compensated by one another or by R-Ras3, suggesting that they have specific non-redundant functions within the CNS.

Given the high degree of homology shared between the R-Ras subfamily and classical Ras members, we sought to investigate whether an absence of R-Ras1 and/or R-Ras2 could be compensated by overexpression of classical Ras members. To this end, we performed RT-qPCR experiments on ONs from control, *R-Ras1^−/−^*, *R-Ras2^−/−^*, and *R-Ras1^−/−^*;*R-Ras2^−/−^* adult mice, showing that neither *K-Ras*, *N-Ras*, nor *H-Ras* expression levels were modified in the absence of R-Ras1 and/or R-Ras2. Specifically, the fold changes in mRNA levels relative to control mice were as follows: for *K-Ras*, control = 1.00 ± 0.14, *R-Ras1^−/−^* = 1.11 ± 0.08, *R-Ras2^−/−^* = 0.91 ± 0.02, and *R-Ras1^−/−^*;*R-Ras2^−/−^* = 1.07 ± 0.04; for *N-Ras*, control = 1.01 ± 0.12, *R-Ras1^−/−^* = 0.98 ± 0.07, *R-Ras2^−/−^* = 1.09 ± 0.16, and *R-Ras1^−/−^*;*R-Ras2^−/−^* = 1.17 ± 0.02; and for *H-Ras*, control = 1.01 ± 0.20, *R-Ras1^−/−^* = 0.96 ± 0.03, *R-Ras2^−/−^* = 0.98 ± 0.13, and *R-Ras1^−/−^*;*R-Ras2^−/−^* = 1.04 ± 0.05 (Figure 1B). These results were further confirmed by Western blot (WB) experiments on ON lysates incubated with a pan-Ras antibody. Again, we observed no significant changes in classical Ras protein levels in the *R-Ras1^−/−^* (102.10 ± 9.45%), *R-Ras2^−/−^* (98.75 ± 10.82%), or *R-Ras1^−/−^*; *R-Ras2^−/−^* (98.64 ± 15.03%) mice relative to controls (Figure 1C,D), suggesting specific functions for these GTPases that could not be compensated by the classical Ras family.

### 2.2. R-Ras1 and R-Ras2 Are Expressed at Different Levels throughout the CNS

**Figure 2:**
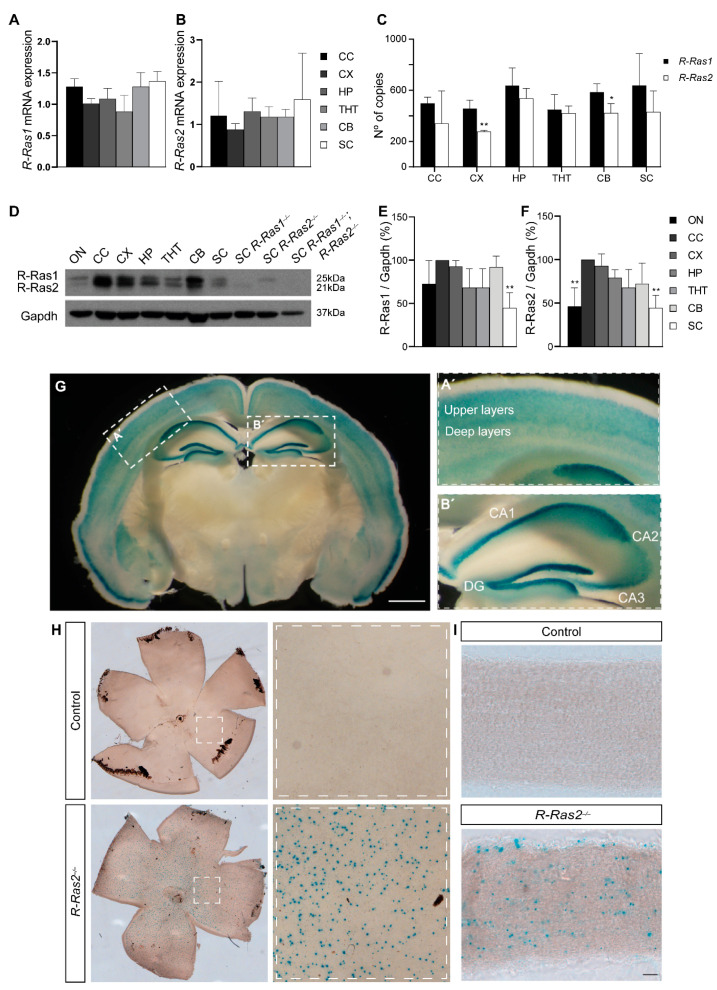
R-Ras1 and R-Ras2 expression in different CNS regions. (**A**) Relative RT-qPCR experiments show relative expression of *R-Ras1* in the CC, CX, HP, THT, CB, and SC of control adult mice (P90); there were no differences in *R-Ras1* mRNA expression across tissues. (**B**) RT-qPCR experiments display relative expression of *R-Ras2* in the CC, CX, HP, THT, CB, and SC of control adult mice (P90); there were no differences in *R-Ras2* mRNA expression across tissues. (**C**) Absolute RT-qPCR experiments show number of mRNA copies of *R-Ras1* and *R-Ras2* in the CC, CX, HP, THT, CB, and SC of control adult mice (P90); *R-Ras2* mRNA expression was significantly lower in the CX (** *p* < 0.01) and CB (* *p* < 0.05) compared to *R-Ras1*. (**D**) Western blots from the ON, CC, CX, HP, THT, CB, and SC lysates of adult (P90) control mice and SC lysates from *R-Ras1^−/−^*, *R-Ras2^−/−^*, and *R-Ras1^−/−^*;*R-Ras2^−/−^* adult mice. (**E**) Quantification of R-Ras1 protein levels normalized to GAPDH revealed significantly lower levels of R-Ras1 in the SC (** *p* < 0.01) relative to the CC. (**F**) Quantification of R-Ras2 protein levels normalized to GAPDH showed significantly lower levels of R-Ras2 in the ON (** *p* < 0.01) and SC (** *p* < 0.01) relative to the CC. (**G**) X-Gal staining on a coronal section from an *R-Ras2^−/−^* mouse displaying those areas where R-Ras2 would be expressed in blue. (**A’**) Higher magnification image of the cortex displaying greater staining in the upper layers. (**B’**) Higher magnification image of the hippocampus displaying greater staining in the CA1, CA2, CA3, and DG. Bar graph represents the mean ± SD of the change as a percentage. (**H**) X-Gal staining on flat-mounted retinas from control and *R-Ras2^−/−^* mice, showing where R-Ras2 would be expressed in blue. (**I**) X-Gal staining on longitudinal optic nerve sections from control and *R-Ras2^−/−^* mice, showing where R-Ras2 would be expressed in blue. A two-tailed Student’s *t*-test was used for statistical analysis. ON: optic nerve; CC: corpus callosum; CX: cerebral cortex; HP: hippocampus; THT: thalamus–hypothalamus; CB: cerebellum; SC: spinal cord; GAPDH: glyceraldehyde 3-phosphate dehydrogenase; SD: standard deviation; CA: cornu ammonis; DG: dentate gyrus. *n* = 3. Scale bars: (**G**), 2 mm; (**H**) 500 μm; (**I**), 75 μm.

Little is known about R-Ras1 and R-Ras2 expression patterns in the CNS. For this reason, we first performed relative RT-qPCR experiments on several CNS anatomical regions of control adult mice, including the corpus callosum (CC), cerebral cortex (CX), hippocampus (HP), thalamus–hypothalamus (THT), cerebellum (CB), and SC. We found that both *R-Ras1* and *R-Ras2* are expressed throughout several tissues of the CNS at similar levels. For *R-Ras1*, the fold change values relative to the CB (1.28 ± 0.22) were 1.28 ± 0.12 in the CC, 1.01 ± 0.79 in the CX, 1.09 ± 0.16 in the HP, 0.89 ± 0.26 in the THT, and 1.37 ± 0.15 in the SC (Figure 2A). On the other hand, the fold change values for *R-Ras2* relative to the CB (1.18 ± 0.17) were 1.21 ± 0.0.81 in the CC, 0.88 ± 0.13 in the CX, 1.31 ± 0.32 in the HP, 1.18 ± 0.24 in the THT, and 1.59 ± 1.09 in the SC (Figure 2B). No significant differences were found in the relative expression of *R-Ras1* or *R-Ras2* across these tissues.

Next, we wanted to investigate whether the expression of *R-Ras1* and *R-Ras2* was different within the same tissue. To this end, we performed absolute RT-qPCR experiments in the CC, CX, HP, THT, CB, and SC of control adult mice. The results revealed that, although the levels of expression of *R-Ras1* and *R-Ras2* were similar in most tissues, *R-Ras1* was expressed more than *R-Ras2* in the CX and CB. Specifically, *R-Ras1* expression values (in copy numbers) were 499 ± 47.3 in the CC, 458 ± 65.3 in the CX, 636 ± 140 in the HP, 450 ± 118 in the THT, 587 ± 66 in the CB, and 639 ± 249 in the SC. In comparison with *R-Ras1*, *R-Ras2* expression values (in copy numbers) were 343 ± 253 in the CC, 278 ± 9.89 in the CX (*p* < 0.01), 539 ± 75.2 in the HP, 421 ± 56.2 in the THT, 423 ± 73.1 in the CB (*p* < 0.05), and 431 ± 164 in the SC (Figure 2C). Taken together, these data show that, in addition to the ON, CC, and SC [30], *R-Ras1* and *R-Ras2* are also expressed in the CX, HP, THT, and CB. Moreover, expression of *R-Ras1* tends to be higher in tissues such as the CX and CB, possibly due to the described expression of R-Ras1 in neurons [32,33,34,35].

To corroborate the expression data obtained from the RT-qPCR assays, we performed WB experiments using the ON, CC, CX, HP, THT, CB, and SC of adult control mice. Given the high homology between R-Ras1 and R-Ras2, most antibodies recognize both proteins. Nevertheless, as previously described by Sanz-Rodriguez et al. [30], both proteins can be specifically identified by their molecular weight: R-Ras1 appears as a band at 25 kDa [35], while R-Ras2 shows a band at 21 kDa [36]. The SC of *R-Ras1^−/−^*, *R-Ras2^−/−^*, and *R-Ras1^−/−^*;*R-Ras2^−/−^* adult mice were used as additional controls to unequivocally recognize each protein. Although no differences were observed at the mRNA level, the WB results revealed significantly lower protein levels of R-Ras1 in the ON and lower protein levels of R-Ras2 in the ON and SC relative to the CC. Specifically for R-Ras1, the fold change values were 72.56 ± 26.96% in the ON, 92.71 ± 6.69% in the CX, 68.26 ± 21.73% in the HP, 68.37 ± 21.59% in the THT, 92.15 ± 12.41% in the CB, and 44.86 ± 17.43% in the SC (*p* < 0.01) relative to controls. For R-Ras2 the fold-change values were 46.49 ± 20.94% in the ON (*p* < 0.01), 92.89 ± 13.38% in the CX, 79.36 ± 8.91% in the HP, 67.87 ± 20.63% in the THT, 72.44 ± 23.59% in the CB, and 44.53 ± 14.53% in the SC (*p* < 0.01) relative to controls (Figure 2C,D).

Given that *R-Ras2^−/−^* mice have a reporter sequence for LacZ that encodes β-galactosidase, we performed X-Gal staining on brain coronal sections of *R-Ras2^−/−^* adult mice (Figure 2E). This assay led us to recognize regions where R-Ras2 was highly expressed. Specifically, we found high levels of R-Ras2 in the CX and HP. In the CX, greater staining was observed in the upper layers, while in the HP reactivity was higher in the CA1 and dentate gyrus. Surprisingly, R-Ras2 expression was higher in less myelinated tissues than in highly myelinated tissues such as the ON or SC. In addition, we studied the presence of R-Ras2 in other anatomical regions of the visual pathway by performing X-Gal staining on whole retinas and longitudinal sections of the ON (Figure 2G,I) of control and *R-Ras2^−/−^* adult mice. The results showed expression of R-Ras2 in the ON and punctate staining on the retinas of *R-Ras2^−/−^* mice, compatible with the RGCs. These results confirm the expression of R-Ras2 in cell types other than OLs.

### 2.3. R-Ras1 and R-Ras2 Are Expressed by Neurons in the CNS, but Not in Astrocytes or Microglia

To finally determine which CNS cell types express R-Ras1 and R-Ras2, we performed double immunostaining against R-Ras1 and R-Ras2 combined with antibodies against microglia, astrocytes, or neurons in coronal sections of areas stained by the X-Gal reaction from *R-Ras1^−/−^* and *R-Ras2^−/−^* adult mice.

**Figure 3:**
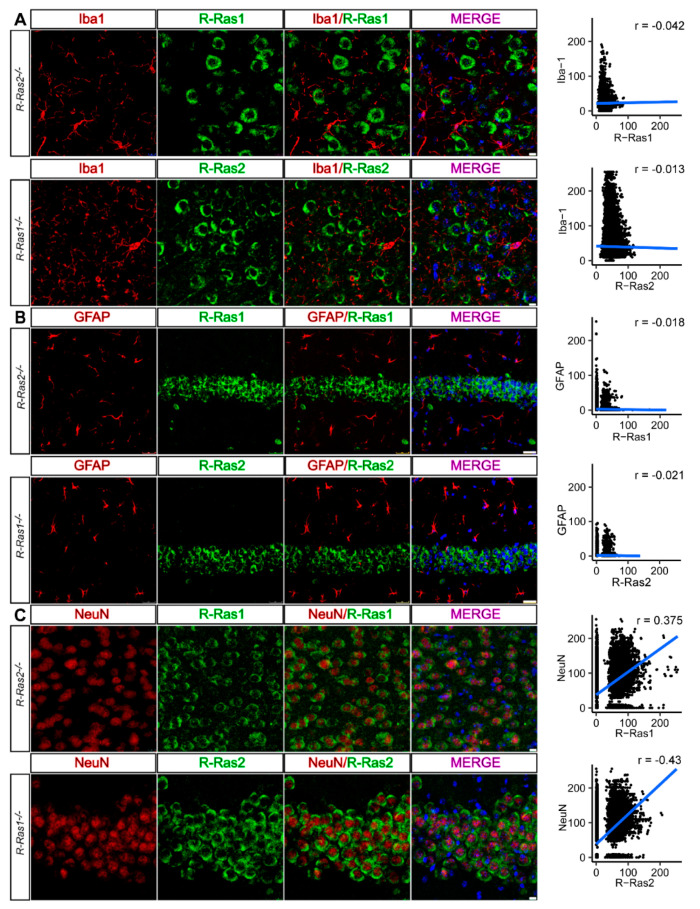
R-Ras1 and R-Ras2 are expressed in neurons, but not in astrocytes or microglia. (**A**) Double immunostaining of coronal sections from *R-Ras1^−/−^* and *R-Ras2^−/−^* adult mice (P90) with antibodies against R-Ras1 or R-Ras2 and the microglial marker Iba1 showed absence of colocalization between both markers. (**B**) Double immunostaining of coronal sections from *R-Ras1^−/−^* and *R-Ras2^−/−^* adult mice (P90) with antibodies against R-Ras1 or R-Ras2 and the astrocyte marker GFAP; neither GTPase colocalized with the GFAP marker. (**C**) Double immunostaining of coronal sections from *R-Ras1^−/−^* and *R-Ras2^−/−^* adult mice (P90) with antibodies against R-Ras1 or R-Ras2 and the neural marker NeuN; both GTPases localized with the NeuN marker within the same cell, indicating that R-Ras1 and R-Ras2 are expressed in neurons. Areas analyzed: dentate gyrus of the hippocampus (ML: 0.5 mm, AP: −2 mm from bregma, DV: 2.2 mm) and upper layers of the somatosensory cortex (ML: 3 mm, AP: −2 mm from bregma, DV: 1.5 mm). Pearson’s r correlation test was used for the statistical analysis. Fluorograms indicate the distribution of pixel intensity values for each marker used. Values similar to 0 or negative indicate the absence of colocalization, while higher values indicate localization within the same cell. Iba1: ionized calcium-binding adapter molecule 1; GFAP: glial fibrillary acidic protein *n* = 4. Scale bars: (**A**,**C**) 10 µm; (**B**) 25 µm.

First, we used an antibody against the specific microglial membrane protein ionized calcium-binding adapter molecule 1 (Iba1) to identify co-expression with R-Ras1 and/or R-Ras2 in the dentate gyrus of the HP (ML: 0.5 mm, AP: −2 mm from bregma, DV: 2.2 mm) and the upper layers of the somatosensory CX (ML: 3 mm, AP: −2 mm from bregma, DV: 1.5 mm). Results showed an absence of colocalization between both markers. Quantification revealed no colocalization between R-Ras1 and Iba1 (r = −0.042) or R-Ras2 and Iba1 (r = −0.013), confirming that neither R-Ras1 nor R-Ras2 are expressed in microglial cells (Figure 3A). The same procedure was followed to identify astrocytes, using glial fibrillary acidic protein (GFAP) as a specific marker. Again, no colocalization was found between GFAP and R-Ras1 (r = −0.018) or R-Ras2 (r = −0.021) (Figure 3B). Lastly, to detect expression of R-Ras1 and/or R-Ras2 in neurons, we used the marker NeuN. We observed that both markers were located within the same cell, consistent with higher Pearson’s correlation values for R-Ras1 and NeuN (r = 0.375), and for R-Ras2 and NeuN (r = 0.43). The results showed that both R-Ras1 and R-Ras2 are expressed in neurons (Figure 3C) of the CX and the dentate gyrus of the HP, which would explain the observed differences in R-Ras expression between the CNS anatomical regions, as well as their degree of myelination. Together, our results show for the first time that R-Ras2 is expressed in neurons.

## 3. Discussion

The Ras family was the first small GTPase group to be discovered, more than 30 years ago, and is particularly involved in development, proliferation, differentiation, and survival [21,24]. Within the R-Ras subfamily, R-Ras3 was long considered to be the most important R-Ras member in the CNS [27,29], while R-Ras1 and R-Ras2 were thought to play more secondary roles. However, recent studies from our laboratory demonstrated in vivo that R-Ras1 and R-Ras2 are expressed in OLs, where they play essential roles in cell survival and differentiation [30]. A loss of R-Ras1 or R-Ras2 resulted in reduced numbers of OLs, with a more dramatic decrease observed in the absence of both GTPases. Furthermore, the mutant mice had reduced numbers of mature OLs, with a concomitant increase in immature OL populations. Therefore, mice lacking R-Ras1 and/or R-Ras2 showed varying degrees of hypomyelination in CNS tracts related to the visual pathway, such as the ON and CC. Analysis of the ON by electron microscopy revealed thinner myelin sheaths in the absence of R-Ras1, and 30% fewer myelinated axons due to the lack of R-Ras2, while an absence of both GTPases resulted in a dramatic hypomyelination, where up to 80% of axons were unmyelinated. As myelin thickness and node and internode length condition the efficacy of neural transmission, we further studied the functional consequences of the absence of R-Ras1 and R-Ras2 on the visual pathway. Consistent with studies where a lack of myelin slowed down impulse transmission [37,38,39], we found reduced conduction velocity in the optic tracts of the single- and double-mutant mice. The findings presented here show the expression of these GTPases in the retina, although their low expression would not explain the dramatic alterations observed in conduction velocity. For this reason, we believe that this effect is mediated by the functional role of R-Ras1 and R-Ras2 in OLs, rather than that in neurons. In future studies, we will analyze the roles of these GTPases in neurons in greater depth.

Our current data demonstrate that R-Ras1 and R-Ras2 have functions that are distinct from those of other R-Ras members and the classical Ras. RT-qPCR and WB studies showed that the absence of R-Ras1 and/or R-Ras2 does not change expression levels of R-Ras3 or the most closely related Ras members. Moreover, we analyzed the expression of R-Ras1 and R-Ras2 in order to explore their potential importance in other CNS anatomical regions, showing them both to be widely expressed throughout all of the brain structures tested. In addition to the previously described expression in the ON, CC, and SC, we found that *R-Ras1* and *R-Ras2* are also expressed at similar levels in the CX, HP, THT, and CB. We further performed absolute RT-qPCR experiments to see whether there were significant differences in the expression of *R-Ras1* and *R-Ras2* within the same tissues. We found that the number of copies of *R-Ras1* was significantly higher than that of *R-Ras2* in the CX and CB, probably due to the fact that R-Ras1 is expressed in neurons other than OLs [32,33,34,35,40]. Although we did not find differences in the relative RT-qPCR assays, WB experiments showed that R-Ras1 and R-Ras2 protein levels vary between tissues. Even though we had expected higher levels of R-Ras1 and R-Ras2 in highly myelinated tissues such as the ON and SC, the results revealed that the ON and SC were actually the tissues with the least R-Ras1 and R-Ras2 expression. It has been described that R-Ras1 is expressed in cortical and hippocampal neurons other than OLs [32,33,34,35,40], which may justify the higher levels of R-Ras1 seen in less myelinated tissues such as the CX or HP. However, higher levels of R-Ras2 were also found in the CX, HP, and retina, suggesting that they may be widely expressed in other cell types that have never been described before. Interestingly, the pattern of expression was higher in the CX and dentate gyrus of the HP, again suggesting that R-Ras2 may be expressed in other cellular types. Finally, confocal microscopic imaging allowed us to disregard expression of R-Ras1 and R-Ras2 in astrocytes and microglia, though we found for the first time that R-Ras2 is expressed in neurons.

Given the close relationship between neurons and OLs in the myelination process, and knowing that R-Ras1 and R-Ras2 are expressed in both cell types, we cannot disregard that an absence of neuronal R-Ras1 or R-Ras2 contributes to the visual deficits observed in mutant mice. Future work is essential in order to segregate the functions that these GTPases may play in each cell type during myelination, pointing us toward a new field studying the role of R-Ras2 in neurons.

## 4. Materials and Methods

### 4.1. Animals

C57BL6 mice were housed in specific pathogen-free conditions in a humidity- and temperature-controlled room with a 12 h light/dark cycle, receiving water and food ad libitum. All experiments were performed in male and female mice, and all animal procedures were approved by the corresponding institutional ethical committee (Centro de Biología Molecular Severo Ochoa, CBMSO, Madrid, Spain) and were performed in accordance with Spanish and European guidelines. All efforts were made to minimize animal suffering.

*R-Ras1^−/−^* mice were generated at genOway (Lyon, France) using the targeting construction BAL1-HR with a neomycin resistance cassette flanked by FRT sequences, inserted in the intron 1 and LoxP sites flanking exons 2 and 6. The construction was electroporated into embryonic stem cells derived from murine 129Sv/Pas and selected by G418 antibiotic. Southern blotting was used to verify correct homologous recombination. Heterozygous mice were crossed, and offspring littermates were genotyped by PCR to ensure that all animals used were homozygous for the knockout (Table 1). PCR was performed on an Applied Biosystems™ 2700 Thermal Cycler (Waltham, MA, USA) as follows: 94 °C for 5 min; 30 cycles of 94 °C for 30 s, 60 °C for 30 s, and 72 °C for 30 s; and a final step of 72 °C for 7 min. Samples were then run on a 2.5% agarose gel in Tris/Borate/EDTA (TBE) buffer at 100 V for 30 min, and visualized in a transilluminator. *R-Ras2^−/−^* mice were generated at Lexicon Pharmaceuticals (The Woodlands, TX, USA), and were derived from embryonic stem cell clone OST361011 with insertion of retroviral VICTR37 in the middle of intron 1 of R-Ras2. Heterozygous mice were crossed and offspring littermates were genotyped by PCR to ensure that all animals used were homozygous for the knockout (Table 1) [36]. The same PCR conditions were used as for the R-Ras1^−/−^ mice.

*R-Ras1^−/−^* and *R-Ras2^−/−^* mice were kindly provided by Professor B. Alarcón (CBMSO). *R-Ras1^−/−^*;*R-Ras2^−/−^* mice were generated by backcrossing individual lines of *R-Ras1^−/−^* and *R-Ras2^−/−^* mice. Offspring littermates were genotyped by PCR to ensure that all animals used were homozygous for both knockout genes. All experiments were performed using wild-type mice (*R-Ras1^+/+^*;*R-Ras2^+/+^*) as controls. Animals were maintained in a C57BL6J background.

### 4.2. Reverse Transcription Quantitative PCR (RT-qPCR)

The different CNS anatomical regions of interest were harvested from adult (P90) control, *R-Ras1^−/−^*, *R-Ras2^−/−^*, and *R-Ras1^−/−^*;*R-Ras2^−/−^* mice after cervical dislocation and decapitation (*n* = 3 per genotype). To preserve RNA, freshly extracted tissue was immediately frozen in TRIzol™ (Thermo Fisher Scientific, Waltham, MA, USA, catalog #15596026) at −80 °C. RNA extraction and purification were performed using an RNeasy Mini Kit (Qiagen, Germantown, MD, USA, catalog #74106), following the manufacturer’s protocol. RNA concentration and integrity were measured using a NanoDrop (Thermo Scientific NanoDrop One) and a Bioanalyzer (Bioanalyzer Agilent 2100, Santa Clara, CA, USA), respectively. Most of the samples showed 260/280 and 260/230 ratios around 2, and RNA integrity values (RIN) of the samples varied between 7.4 and 9.5. Complementary DNA (cDNA) was generated from 500 ng of total ON RNA using the iScript cDNA Synthesis Kit (Bio-Rad, Hercules, CA, USA, catalog #1708891) in a 20 μL final reaction volume. SsoFast EvaGreen Supermix (Bio-Rad, Hercules, CA, USA, catalog #1725204) reagent was used for qPCR expression profiling, carried out on a CFX384 Real-Time System C1000 Thermal Cycler (Bio-Rad, Hercules, CA, USA) in hard-shell 384-well PCR plates (White Well Clear Shell; Bio-Rad, Hercules, CA, USA, catalog #HSP3805). A total of 5 ng of cDNA per sample was used in a 10 μL final volume reaction. Technical triplicates were performed in order to correct for pipetting errors in plate loading. No-template control (NTC) reactions were carried out using all reagents except the sample to disregard potential contamination. ValidPrime assay was performed to control the presence of genomic DNA background signals during qPCR expression profiling [41]. Relative quantification was performed using the 2^ΔΔCq^ method [42], taking *GAPDH, ActB, Hprt1, Tbp, Arbp*, and *GusB* as reference genes for sample normalization. The gene expression levels in control groups were normalized to 1. For absolute quantification experiments, standard curves were performed with a qPCR over an eight-point ¼ dilution curve made from 1.56 × 10^5^ copies/μL of R-Ras1 or R-Ras2. Samples were normalized to the expression levels of *GusB* and *Arbp*. The RT-qPCR primers used in this study can be found in Table 2.

### 4.3. Western Blotting

Tissue samples (ON, CC, CX, HP, THT, CB, and SC) from adult (P90) mice were dissected and resuspended in lysis buffer (50 mM Tris pH 8.0, 150 mM NaCl, 1% NP40, 2 mM EDTA, 0.1% SDS, 0.5% deoxycholate, and protease inhibition mixture; Roche, Basel, Switzerland, 11697498001) and phenylmethylsulfonyl fluoride (PMSF). Lysates were denatured by boiling them for 5 min in protein-loading buffer (50 mM Tris–HCl pH 6.8, 2% SDS, 10% glycerol, 1% β-mercaptoethanol (BME), 12.5 mM EDTA, and 0.02% bromophenol blue) and resolved in 10–12% SDSP gels in the presence of BME. Gels were run at constant current starting at 90 or 100 V. After electrophoresis, samples were transferred by electroblotting onto a polyvinylidene difluoride (PVDF) membrane in a semidry electroblotting system (Trans-Blot Turbo. Bio-Rad, Hercules, CA, USA) at 1.2 mA/cm^2^ for 35–40 min. Nonspecific protein binding was blocked by incubating the membrane with 5% non-fat milk in TBS-Tween-20 for 1 h at room temperature. Membranes were incubated overnight with the pertinent primary antibodies in the blocking buffer (Table 3). After washing, blots were incubated for 1 h with appropriated peroxidase-conjugated secondary antibodies (Table 3). Labeled proteins were detected with the chemiluminescence reagent ECL (GE Healthcare, Chicago, IL, USA). Densitometric analysis was performed using a GS-800 Calibrated Densitometer (Bio-Rad, Hercules, CA, USA).

### 4.4. X-Gal Reaction

X-Gal staining was performed on fresh, non-perfused brains from R-Ras2^−/−^ mice following widely established protocols [43]. After cervical dislocation, brains were extracted, washed in 1× PBS, and mounted in a solution of 10% sucrose and −4% agarose to increase tissue robustness. Then, they were sectioned on a Leica VT-1200S sliding-blade vibratome to produce 300–400 μm thick sections that were stained with X-Gal reactive at 37 °C for 4 h or overnight. During this reaction, X-Gal (5-bromo-4-chloro-3-indolyl-β-D-galactopyranoside) was hydrolyzed by the β-galactosidase present in the cassette of R-Ras2^−/−^ mice to form galactose and 5-bromo-4-chloro-3-hydroxyindole—an insoluble blue compound. Tissues were later fixed in 4% paraformaldehyde (PFA) and photographed on a Leica MZ6 magnifying glass.

### 4.5. Immunohistochemistry

Animals were anesthetized (ketamine 100 mg/kg and xylazine 10 mg/kg intraperitoneally) and perfused transcardially with 0.1 M PBS (pH 7.4) followed by 4% PFA in PBS. Perfused tissues were removed and postfixed in 4% PFA at 4 °C overnight. Then, they were cryoprotected in 30% sucrose in PBS and embedded and frozen in a 7.5% gelatin in 15% sucrose solution. Then, they were sectioned on a cryostat to produce 20 μm cryosections on SuperFrost Plus microscope slides (Thermo Fisher Scientific, Waltham, CA, USA, catalog # 22-037-246). Sections were blocked for 1 h at room temperature with 10% fetal bovine serum in PBS containing 0.5% Triton-X 100 (blocking solution), and then incubated overnight at 4 °C with the pertinent primary antibodies (Table 4). After 3 washes, fluorescent-tagged secondary antibodies (Table 4) were applied for 1 h at room temperature, and sections were counterstained with DAPI (Sigma-Aldrich, St. Louis, MO, USA, catalog# 32670) and mounted in Aqua-Poly/Mount mounting medium (PolySciences, Warrington, PA, USA, catalog# 18606).

### 4.6. Confocal Microscopy

Fluorescence images were obtained using a confocal multispectral Leica TCS SP5 system (Leica Microsystems, Wetzlar, Germany) controlled by Las AF v 2.7 software (Leica). Image acquisition was performed sequentially using a 40×/1.4 NA oil immersion objective and appropriate fluorochrome excitation lines (405 nm, 488 nm, and 560 nm for DAPI, Alexa-488, and Alexa-555, respectively).

### 4.7. Statistical Analysis

Quantitative data are shown as the mean ± SD. The experimental groups were compared using a two-tailed Student’s *t*-test. Statistical numeric data are provided in the figure legends. (*): *p* < 0.05; (**): *p* < 0.01; (***): *p* < 0.001. An alpha level of 0.05 was considered significant. Statistical analysis was performed using GraphPad Prism 8 statistical software.

## Figures and Tables

**Table 1:** PCR primer sequences used for murine colony genotyping. Primer sequences are stated from 5′ to 3′.

Target	Forward Primer	Reverse Primer	Size ^1^
*R-Ras1^+/+^*	CGCTCTAGCTGAGCCTCTGT	TACAGGGTCTTGTGGGGAAA	138
*R-Ras2^+/+^*	TGAAACAGGATCATGTTGTGGAG	CAGGAGGAGTCCAAGAAGAC	266
*R-Ras1^−/−^*	GGAGCAAGAGGAGGGAAGGAATG	CTTCGAGAGGACTCAGTTCAATCC	1000
*R-Ras2^−/−^*	TGAAACAGGATCATGTTGTGGAG	ATAAACCCTCTTGCAGTTGCATC	128

^1^ Amplicon size is stated in base pairs.

**Table 2:** Primer sequences used for RT-qPCR. Primer sequences are stated from 5′ to 3′.

Target	Forward Primer	Reverse Primer	Size ^1^
*R-Ras1*	TCACAAGCTGGTGGTCGTAG	TGGGATCATAGTCAGACACAAAG	95
*R-Ras2*	CGTGATGAGTTTCCCATGATT	TAACTGCTGCCCTTCTTCCT	87
*R-Ras3*	TGGGCCATCTTGGATGTT	CTGTGCGCATGTATTGTTCC	76
*K-Ras*	TGTGGATGAGTATGACCCTACG	CCCTCATTGCACTGTACTCCT	122
*N-Ras*	GAACTGGCCAAGAGTTACGG	TGTAAAAGGCATCCTCCACA	79
*H-Ras*	CGCCAGCAAGCGGTG	GGTAGGAGTCCTCTATAGTGGGATCA	174
*ActB*	CTAAGGCCAACCGTGAAAAG	ACCAGAGGCATACAGGGACA	104
*Arbp*	GATGCCCAGGGAAGACAG	TCCAAAAGTTGGATGATCTTGA	66
*Gapdh*	CACCACCAACTGCTTAGCCC	TGTGGTCATGAGCCCTTCC	76
*GusB*	AGCCGCTACGGGAGTCG	GCTGCTTCTTGGGTGATGTCA	76
*Hprt1*	TCCTCCTCAGACCGCTTTT	CCTGGTTCATCATCGCTAATC	90
*Tbp*	CCACAGGGCGCCATGA	GCTGTGGAGTAAGTCCTGTGCC	76

^1^ Amplicon size is stated in base pairs.

**Table 3:** Primary and secondary antibodies used for Western blot experiments.

Target	Host	Dilution	Manufacturer	Catalog	RRID
GAPDH	Mouse	1:1000	Santa Cruz Biotechnology	Sc-365062	AB_10847862
Pan-Ras	Mouse	1:300	Millipore	OP40	AB_213400
R-Ras1	Rabbit	1:200	Abcam	Ab154962	AB_2894924
R-Ras2	Rabbit	1:200	Professor B. Alarcón (CBMSO) [36]		AB_2895064
Anti-Mouse	Goat-HRP	1:5000	Santa Cruz Biotechnology	Sc-2005	AB_631736
Anti-Rabbit	Goat-HRP	1:5000	Southern Biotech	4030-05	AB_2687483

**Table 4:** Primary and secondary antibodies used for immunohistochemistry experiments.

Target	Host	Dilution	Manufacturer	Catalog	RRID
GFAP	Mouse	1:1000	Sigma-Aldrich	G3893	AB_477010
Iba-1	Mouse	1:1000	Millipore	MABN92	AB_10917271
NeuN	Mouse	1:500	Millipore	MAB377	AB_2298772
R-Ras1	Rabbit	1:200	Abcam	Ab154962	AB_2894924
R-Ras2	Rabbit	1:200	Professor B. Alarcón (CBMSO)		
Anti-Mouse	Donkey-Alexa488	1:500	Thermo Fisher	A-21202	AB_141607
Anti-Rabbit	Donkey-Alexa555	1:500	Thermo Fisher	A-31572	AB_162543

## Data Availability

The data that support the findings of this study are available upon reasonable request.
